# Urbanization is Associated with Increased Trends in Cardiovascular Mortality Among Indigenous Populations: the PAI Study

**DOI:** 10.5935/abc.20180026

**Published:** 2018-03

**Authors:** Anderson da Costa Armstrong, Ana Marice Teixeira Ladeia, Juracy Marques, Dinani Matoso Fialho de Oliveira Armstrong, Antonio Marconi Leandro da Silva, Jeová Cordeiro de Morais Junior, Aldina Barral, Luis Claudio Lemos Correia, Manoel Barral-Netto, João A. C. Lima

**Affiliations:** 1 Universidade Federal do Vale do São Francisco (UNIVASF), Petrolina, PE - Brazil; 2 Escola Bahiana de Mediciana e Saúde Pública, Salvador, BA - Brazil; 3 Universidade do Estado da Bahia (UNEB) , Salvador, BA - Brazil; 4 Centro de Pesquisas Gonçalo Moniz da Fundação Oswaldo Cruz (FIOCRUZ), Salvador, BA - Brazil; 5 Johns Hopkins University, Baltimore - USA

**Keywords:** Indigenous Population, Cardiovascular Diseases / mortality, Urbanization / trends, Social Change

## Abstract

**Background:**

The cardiovascular risk burden among diverse indigenous populations is not totally known and may be influenced by lifestyle changes related to the urbanization process.

**Objectives:**

To investigate the cardiovascular (CV) mortality profile of indigenous populations during a rapid urbanization process largely influenced by governmental infrastructure interventions in Northeast Brazil.

**Methods:**

We assessed the mortality of indigenous populations (≥ 30 y/o) from 2007 to 2011 in Northeast Brazil (Bahia and Pernambuco states). Cardiovascular mortality was considered if the cause of death was in the ICD-10 CV disease group or if registered as sudden death. The indigenous populations were then divided into two groups according to the degree of urbanization based on anthropological criteria:^9,10^ Group 1 - less urbanized tribes (Funi-ô, Pankararu, Kiriri, and Pankararé); and Group 2 - more urbanized tribes (Tuxá, Truká, and Tumbalalá). Mortality rates of highly urbanized cities (Petrolina and Juazeiro) in the proximity of indigenous areas were also evaluated. The analysis explored trends in the percentage of CV mortality for each studied population. Statistical significance was established for p value < 0.05.

**Results:**

There were 1,333 indigenous deaths in tribes of Bahia and Pernambuco (2007-2011): 281 in Group 1 (1.8% of the 2012 group population) and 73 in Group 2 (3.7% of the 2012 group population), CV mortality of 24% and 37%, respectively (p = 0.02). In 2007-2009, there were 133 deaths in Group 1 and 44 in Group 2, CV mortality of 23% and 34%, respectively. In 2009-2010, there were 148 deaths in Group 1 and 29 in Group 2, CV mortality of 25% and 41%, respectively.

**Conclusions:**

Urbanization appears to influence increases in CV mortality of indigenous peoples living in traditional tribes. Lifestyle and environmental changes due to urbanization added to suboptimal health care may increase CV risk in this population.

## Introduction

The urbanization process is a concern in developing countries, as it influences the prevalence of cardiovascular (CV) risk factors and coronary disease.^1^ In fact, an early process of lifestyle changes appears to lead to increases in CV risk when rural migrants settle in metropolitan areas.^2^ Moreover, traditional indigenous populations are recognized as in greater risk of CV complications.^3^

Diverse infectious diseases caused major health concerns when Europeans initially contacted Native American indigenous populations. Along the years, a shift in indigenous mortality rates has been shown toward chronic diseases affected by lifestyle changes, which varies highly across diverse native populations.^4-6^ In recent years, isolated indigenous people in Brazil still showed low blood pressure that appears to be related to their traditional lifestyle.^7,8^

Major infrastructural projects may rapidly influence populations in the surrounding areas, often affecting indigenous communities. More recently, the Sao Francisco Valley in Northeast Brazil has been experiencing major changes in infrastructure - particularly regarding construction of large dams and canals - that appear to affect traditional indigenous lifestyle in the area.^9,10^ It is unclear, however, how the urbanization process has been affecting CV mortality in native indigenous communities over the years.

The Project of Atherosclerosis Among Indigenous populations (PAI) was created to investigate the impact of urbanization on CV diseases among indigenous communities in the Sao Francisco Valley (Northeast Brazil). In this study, we investigate the CV mortality profile of indigenous populations during a rapid urbanization process that was largely influenced by governmental infrastructure interventions in the Sao Francisco Valley. For this purpose, we assessed longitudinal data on mortality rates of indigenous and non-indigenous populations in different degrees of urbanization.

## Methods

### Study population

We assessed data for indigenous mortality in the Sao Francisco Valley, Northeast Brazil (states of Bahia and Pernambuco) between 2007 and 2011, excluding deaths under the age of 30 years. We also assessed the total population in the Sao Francisco Valley according to the Brazilian Institute of Geography and Statistics.

The indigenous populations were then divided into two groups according to the degree of urbanization based on previous anthropological evaluations:^9,10^ Group 1 - less urbanized tribes (Funi-ô, Pankararu, Kiriri, and Pankararé); and Group 2 - more urbanized tribes (Tuxá, Truká, and Tumbalalá).

We also assessed the mortality for the total population in two important and highly urbanized cities in the Sao Francisco Valley: Juazeiro and Petrolina. The Sao Francisco Valley University Ethics Committee approved this study.

### Mortality data

The Brazilian Indigenous Healthcare Subsystem is currently the responsibility of the Special Secretariat of Indigenous Health, a section of the Ministry of Health, which, since 2007, has implemented a surveillance program regarding mortality.^11,12^ Indigenous mortality was assessed from the official records of the Special Secretariat of Indigenous Health. Mortality in the largest cities of the Sao Francisco Valley used the Brazilian Health Ministry registry (DATASUS/TABNET: http://datasus.saude.gov.br/). Mortality was classified according to the ICD-10 groups. Cardiovascular mortality was considered if the cause of death was in the ICD-10 CV disease group or if registered as sudden death.

### Statistical analysis

An exploratory analysis was performed to show trends of CV mortality in diverse indigenous populations over time. Trends over the years in CV mortality in adults (≥ 30 y/o) were shown as the percentage of the total deaths at the same age range for total indigenous communities in the Sao Francisco Valley and according to the urbanization group (less urbanized tribes in Group 1, more urbanized tribes in Group 2, and highly urbanized cities). Two Sample Test for Proportions assessed differences in CV mortality rates among indigenous populations. Statistical significance was established if p value < 0.05. STATA 10 was used for computing statistics.

## Results

A total of 75,635 people was registered as indigenous in the Special Indigenous Health Districts of Bahia and Pernambuco. Of these, 25,560 were living in the assessed tribes of the Sao Francisco Valley, mostly in the less urbanized Group 1 tribes (Table 1).

**Table 1:** Description of indigenous populations in the Sao Francisco River Basin, according to the study groups.

Groups	Ethnicity	Population^¥^	Villages	Total deaths*
Group 1	Funi-ô	4,564	7	58
	Pankararu	7,650	27	161
	Kiriri	2,185	15	36
	Pankararé	1,535	11	26
	TOTAL	15,934		281
Group 2	Tuxá	1,665	11	26
	Truká	6,741	36	39
	Tumbalalá	1,220	8	8
	TOTAL	9,626		73

¥ As registered by the Brazilian Institute of Geography and Statistics for 2012;

* Deaths of indigenous people ≥ 30 years old, between 2007 and 2011.

**Figure 1:**
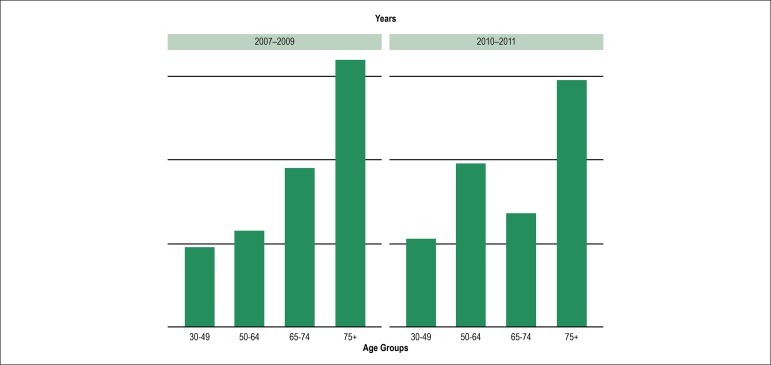
Mortality distribution for indigenous communities in the Sao Francisco Valley (Northeast Brazil) according to age groups.

There was a tendency for mortality at a younger age between 2010 and 2011 when compared to 2007-2009 (Figure 1).

The total of 1,333 deaths was registered for adult indigenous people in the Sao Francisco Valley, 281 deaths (1.8% of the population in 2012) in Group 1 (less urbanized) and 73 deaths (3.7% of the population in 2012) in Group 2 (more urbanized). Between 2007 and 2009, there were 133 deaths in Group 1 and 44 total deaths in Group 2. Between 2009 and 2010, there were 148 total deaths in Group 1 and 29 deaths in Group 2. Table 1 shows the absolute number of deaths in the indigenous people of the Sao Francisco Valley according to the study groups.

**Figure 2:**
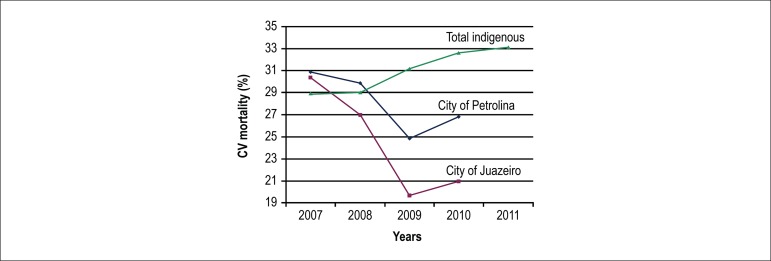
Cardiovascular mortality (≥ 30 y/o) in indigenous and urban populations in the Sao Francisco Valley (Northeast Brazil). Total indigenous refers to total deaths among indigenous populations in the Sao Francisco Valley, Northeast Brazil.

The proportion of CV mortality has shown consistent increases along time in the assessed populations. Conversely, CV mortality has shown consistent decreases for the largest cities in the Sao Francisco Valley (Figure 2).

**Figure 3:**
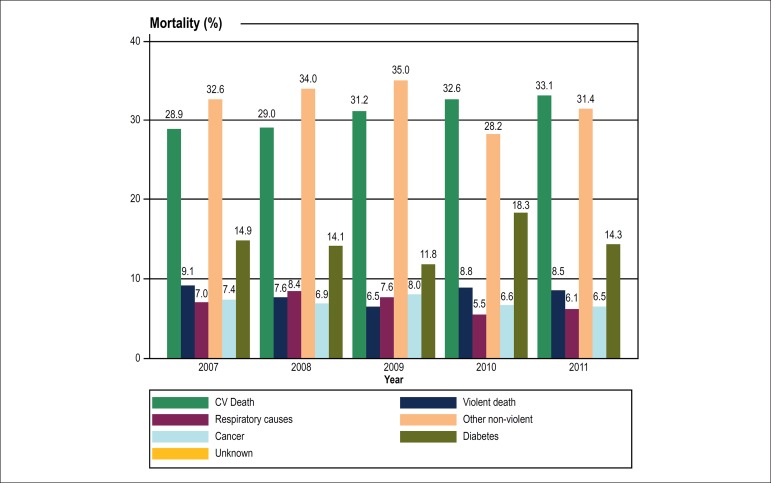
Mortality (≥ 30 y/o) in indigenous populations in the Sao Francisco Valley (Northeast Brazil), according to the degree of urbanization. Group 1 - less urbanized tribes; Group 2 - more urbanized tribes according to anthropological criteria.

When the degree of urbanization was considered for the entire period of observation, CV mortality rates were 24% and 37% in Group 1 and Group 2, respectively (p = 0.02). We also found a trend toward a steeper increase in Group 2 CV mortality along time, while Group 1 had nearly stable proportions of CV deaths (Figure 3).

## Discussion

For the first time in the literature, we show indigenous mortality in the Sao Francisco Valley (Northeast Brazil) tending to a younger age over time, with increasing trends in the proportion of CV deaths. Increases in CV mortality rates in indigenous people living in an area of rapid infrastructural development may indicate that these populations are in harm's way due to changes related to the urbanization process. The knowledge of CV risk and mortality may aid in health policy planning for endangered traditional indigenous populations.

We assessed the available mortality rates - usually a reliable source of information - to explore the indigenous CV burden in Northeast Brazil's Sao Francisco Valley. This area has been through accelerated infrastructural development, such as construction of large canals and dams. Along recent year, hydroelectric power plants have been constructed along the Sao Francisco River, which now the highest concentration of power plants in Brazil.^9^ Our findings indicate that the traditional indigenous populations affected by a rapid urbanization process are at increased risk of CV mortality.

Urbanization may be related to CV risk beyond ethnicity. In this regard, African Americans have shown higher coronary heart disease mortality rates than Whites, but apparently there are additional disparities according to the urbanization level of the population. The coronary disease-related mortality rates in large metropolitan areas showed a decline over the years in a higher magnitude compared to rural areas.^13^ Similar findings have been reported in diverse countries.^14-16^ There are few reports on indigenous health in Brazil, but surveys suggest that indigenous people have a less favorable CV risk profile than the general population.^17,18^ Importantly, lifestyle differences related to CV risk are found in closely related traditional communities.^19^ In fact, rapid changes in lifestyle affect indigenous populations differently from people in urban areas.^20^

Not only risk factors appear to be increasing among indigenous people; the complications related to health care quality are also alarming. In fact, there is evidence that urbanization directly affects the health care quality of a given area.^21^ Additionally, socioeconomic disadvantages do not seem to completely explain the increasing CV risk trends in indigenous populations. Regions majorly populated by indigenous people show increased CV risk beyond the effects of socioeconomic disadvantage.^3,22^ This may be related to difficulties for indigenous populations when interacting with other ethnicities regarding their traditional medicine.^23^

The classic expected dynamics of epidemiology for indigenous people in Brazil was based on two initial steps more closely related to infectious diseases, and a third step of epidemiologic transition and cultural losses. This third period would be characterized by an increase in chronic conditions such as CV disease and the emergence of an epidemiological profile similar to that of non-indigenous communities.^24^ Our findings suggest that an epidemiological fourth step may be underway, in which the occurrence of CV diseases among indigenous people is not similar to that of the general population, but higher. These findings may be explained by rapid lifestyle and environmental modifications, added to a lower health care quality.

Our study had several limitations and should be interpreted in the context of an exploratory investigation. Furthermore, we were limited to assessing the increases in the profile of CV risk factors as we assessed secondary data for mortality. Thus, concerns regarding potential misclassification bias certainly apply. Although large infrastructural changes have historically affected indigenous lifestyles, the magnitude of the deleterious impact of urbanization on the CV risk profile of these groups is not totally clear. Increases in blood pressure, obesity, and glycemic abnormalities are examples of known CV risk factors that may lead to subclinical cardiac abnormalities over time, before a CV event is established.^25-27^ Further studies in the context of the PAI project are planned to address early subclinical abnormalities in these populations.

## Conclusions

In conclusion, we show increasing trends in CV mortality over time among indigenous populations in the Sao Francisco Valley (Northeast Brazil), which appear to be negatively affected by a higher degree of urbanization. Lifestyle and environmental changes due to urbanization added to suboptimal health care may be implicated in the increase in CV risk among indigenous people.
